# ﻿On *Caledromusrobinsmithi*, a new genus and species of Psychrodromini Martens, 2001 (Crustacea, Ostracoda, Herpetocypridinae) from New Caledonia (Pacific Ocean)

**DOI:** 10.3897/zookeys.1165.104045

**Published:** 2023-06-01

**Authors:** Koen Martens, Vitor Góis Ferreira, Nadiny Martins de Almeida, Janet Higuti

**Affiliations:** 1 Royal Belgian Institute of Natural Sciences, Freshwater Biology, Vautierstraat 29, B-1000 Brussels, Belgium; 2 Ghent University, Dept. Biology, K.L. Ledeganckstraat 35, B-9000 Ghent, Belgium; 3 State University of Maringá (UEM), Centre of Biological Sciences (CCB), Department of Biology (DBI), Graduate Program in Ecology of Inland Water Ecosystems (PEA), Av. Colombo, 5790, CEP 87020-900, Maringá, PR, Brazil; 4 State University of Maringá (UEM), Centre of Biological Sciences (CCB), Centre of Research in Limnology, Ichthyology and Aquaculture (Nupélia), Av. Colombo, 5790, CEP 87020-900, Maringá, PR, Brazil

**Keywords:** Chaetotaxy, Cyprididae, hemipenis internal anatomy, morphology, taxonomy

## Abstract

The New Caledonian Archipelago is a hot spot for biodiversity and endemism. Whereas popular groups such as birds and plants are well-studied, invertebrate groups such as ostracods remain ill-known. Here, *Caledromusrobinsmithi***gen. et sp. nov.** is described from a single locality on ‘Grande Terre’, the main island of the archipelago. The new genus belongs to the Psychrodromini, one of the four tribes in the subfamily Herpetocypridinae (family Cyprididae). *Caledromus***gen. nov.** can be distinguished from all other herpetocypridinids by a combination of the following factors: the absence of marginal septa in both valves, the mildly developed marginal valve structures, the small Rome organ on the A1, the total reduction of the five natatory setae on the A2, the rectangular second palp segment of the Mx1, the broad and asymmetrical palp on the female T1, the absence of additional postlabyrinthal coils in the Hp and the seta Sp of the CR which is a fixed spine. Because of the close similarity to the genus *Psychrodromus*, the new genus is thought to have Palaearctic affinities, contrary to other ostracod species in New Caledonia, which are either circumtropical or with Australian zoogeographical connections.

## ﻿Introduction

The New Caledonian archipelago, situated in the Pacific Ocean, north-east of Australia, has high levels of endemic biodiversity, which has attracted the attention of botanists, zoologists and biogeographers alike (Grandcolas 2017). The taxonomy and ecology of larger organisms, such as higher plants (Morat 1993), birds (Dutson 2011) and even freshwater molluscs (Haase and Bouchet 1998) and Trichoptera (Johanson and Ward 2009; Johanson 2017), are relatively well-known. Smaller invertebrate groups, such as ostracods, on the other hand, were largely overlooked in the past and only 16 species were thus far formally reported from this archipelago (mainly by Méhes 1939, but see Kisseih et al. 2020 and references therein in Table 1).

**Table 1.** Present taxonomy of the Herpetocypridinae Kaufmann, 1900.

**Tribe Herpetocypridini** Kaufmann, 1900
*Candonocypris* Sars, 1896
*Herpetocypris* Brady & Norman, 1889
*Ilyodromus* Sars, 1894
*Paranacypris* Higuti, Meisch & Martens, 2009 (change of position)
*Thaicypris* Savatenalinton, 2021
Additional useful (recent) references: Gonzalez Mozo et al. (1996)
Shearn et al. (2014, 2017a, b)
**Tribe Isocypridini** Rome, 1965
*Amphibolocypris* Rome, 1965
*Isocypris* G.W. Müller, 1908
*Platycypris* Herbst, 1957
Additional useful (recent) references: Jocque et al. (2010), Shearn et al. (2014).
**Tribe Psychrodromini** Martens, 2001
***Caledromus* gen. nov**.
*Humphcypris* Martens, 1997
*Psychrodromus* Danielopol & McKenzie, 1977
*Somalicypris* Martens, 1997
Additional useful (recent) references: Baltanás et al. (1993), Zaibi et al. (2013).
**Tribe Stenocypridini** Ferguson, 1964
*Acocypris* Vávra, 1895
*Ampullacypris* De Deckker, 1981
*Chrissia* Hartmann, 1957
*Stenocypria* G.W. Müller, 1901
*Stenocypris* Sars, 1889
Additional useful (recent) references: Savatenalinton and Suttajit (2016),
Moonchaisook and Savatenalinton (2020), Scharf et al. (2020).

For three years (2016–2018), the Muséum national d’Histoire naturelle (Paris, France) organised the New Caledonia Hydrobiological expeditions under the “Our Planet Reviewed/ La Planète revisitée” programme. Two of the authors (JH and KM) participated in these expeditions and collected more than 350 samples from a variety of water bodies and have found close to 50 species of living non-marine Ostracoda of which about half are expected to be new to science. Thus far, *Cyprinotusdrubea*Martens et al., 2019 (subfamily Cyprinotinae Bronshtein, 1947) and *Strandesiamehesi*Kisseih et al., 2020 (subfamily Cypricercinae McKenzie, 1971) were described as new species from these collections.

The subfamily Herpetocypridinae Kaufmann, 1900 is one of the most speciose of the 25 subfamilies in the family Cyprididae Baird, 1845 (Meisch et al. 2019; Savatenalinton 2022a, b). It comprises close to 160 species in 17 genera and four tribes (Table 1). Martens (2001) recognised 11 genera in three tribes. Since then, the genera *Paranacypris*Higuti et al., 2009, *Thaicypris* Savatenalinton, 2021 and presently *Caledromus* gen. nov. have been added, while Shearn et al. (2014) transferred the Isocypridinae Rome, 1965 as a fourth tribe, Isocypridini, into the Herpetocypridinae and as such added three further genera to this subfamily: *Amphibolocypris* Rome, 1965, *Isocypris* G.W. Müller, 1908 and *Platycypris* Herbst, 1957.

Here, we describe a new genus and species, *Caledromusrobinsmithi* gen. et. sp. nov. of the subfamily Herpetocypridinae Kaufmann, 1900 from New Caledonia.

## ﻿Materials and methods

### ﻿Study area

**Figure 1.**
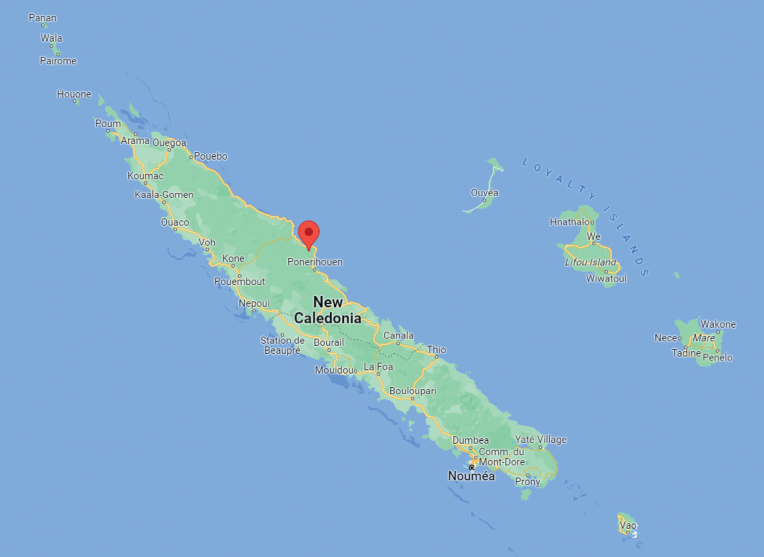
Map showing position of the type locality of *Caledromusrobinsmithi* gen. et sp. nov. on Grande Terre, the main island of the New Caledonian archipelago. Source: Google Maps.

New Caledonia is an archipelago in the south-west Pacific. It is located 1500 km to the north of New Zealand and 1500 km to the east of Australia (Fig. 1). It comprises the main Island “Grande Terre”, the Loyalty Islands (Maré, Lifou, Tiga, and Ouvéa) and other smaller Islands, such as Ile des Pins and Ile Belep. Grande Terre, from which the samples for the current study were obtained, represents the emergent part of the Pacific Norfolk Ridge. New Caledonia lies just north of the Tropic of Capricorn within latitudes 18° and 23° south and longitudes 158° and 172° east (Rawling 2009).

The present material was collected by JH and KM during the 2018 expedition to New Caledonia.

Ostracods were collected by moving a rectangular hand net (28 × 14 cm, mesh size ~ 160 μm) either over sediment (to whirl up the top layers with living biota) or amidst aquatic vegetation. We measured pH (VWR/pH/1100H) and electrical conductivity (EC) / water temperature (VWR/CO/3100H) *in situ*. The position of the type locality on the Grand Terre is illustrated in Fig. 1.

Soft parts were separated from the valves using dissection needles and were then put in a drop of glycerine for the dissection of the appendages. The dissection was covered with a coverslip and sealed with transparent nail polish. Valves were stored dry in micropaleontological slides. Drawings of soft parts were made using a camera lucida (Olympus U-DA) attached to the microscope (Olympus CX-41). Carapace and valves were illustrated and measured using Scanning Electron Microscopy (SEM, Fei Qanta 200 ESEM, in the Royal Belgian Institute of Natural Sciences, Brussels, Belgium) in different views and details.

Type material is lodged in the Muséum national d’Histoire naturelle (**MNHN**, Paris, France) and in the Royal Belgian Institute of Natural Sciences (**RBINS**, Brussels, Belgium).

Chaetotaxy of the limbs follows the model proposed by Broodbakker and Danielopol (1982), revised for the A2 by Martens (1987) and Scharf et al. (2020). Names for the limbs were used according to Meisch (2000) except for caudal ramus, which follows Meisch (2007). Higher taxonomy of the Ostracoda follows Horne et al. (2002) and Meisch et al. (2019).

### ﻿Abbreviations used in text and figures

**Valves and carapaces**:

**Cp** carapace;

**CpD** carapace in dorsal view;

**CpRL** carapace in right lateral view;

**CpV** carapace in ventral view;

**H** height of valves;

**L** length of valves;

**LVi** left valve in internal view;

**RVi** right valve in internal view;

**W** width of valves.

**Limbs**:

**a, b, d** setae on T1;

**A1** antennula;

**A2** antenna;

**alfa, beta, and gamma** setae on Md-palp;

**Att** attachment of the CR;

**CR** caudal ramus;

**d_1_, d_2_, d_p_, e, f, g, h_1_, h_2_, h_3_** setae on T2 and T3;

**db, vb** distal branches of Att;

**ds1, ds2** lobes of distal shield of Hp;

**g, t1-4, z1-3** setae on female A2;

**G1-3, Gm, GM** claws on female A2;

**Ga, Gp, Sa, Sp** claws and setae on the CR;

**Hp** hemipenis;

**Lpp** Left Prehensile palp;

**Md** mandibula;

**Md-palp** mandibular palp;

**M/F** Male/Female;

**ms** medial shield on Hp;

**Mx1** maxillula;

**T1** first thoracopod;

**T2** second thoracopod;

**T3** third thoracopod;

**R** Rome Organ;

**Rpp** Right Prehensile Palp;

**Y, Ya, y3** aesthetascs.

## ﻿Results

**Class Ostracoda Latreille, 1802**

**Subclass Podocopa G.O. Sars, 1866**

**Order Podocopida G.O. Sars, 1866**

**Suborder Cypridocopina Baird, 1845**

**Superfamily Cypridoidea Baird, 1845**

### ﻿Family Cyprididae Baird, 1845

#### ﻿Subfamily Herpetocypridinae

Taxon classificationAnimaliaPodocopidaCyprididae

Kaufmann, 1900

20C7C44A-B501-559F-A9CF-4EF64A2EE53B

##### Diagnosis

**(adapted from Martens 1997).** Large (1.0–3.5 mm), mostly elongated and laterally compressed Cp; marginal valve structures mostly well developed; branched pore canals and marginal septa present in some genera. Most (all?) genera with conical inclusions in the valves, visible with transmitted light as small extra pores. Anterior calcified lamella wider than posterior one; pore-canals mostly branched along ventral margin, simple and straight along anterior and posterior margins; false pore canals present (remnants of fused selvage); selvage and inner lists present or absent.

Al in some genera with large, multi-segmented Rome organs. A2 in males with larger claw Gm developed into a comb-like structure, with one row of strong teeth. C well-developed, symmetrical or asymmetrical; Att of the CR with a triangular basal reinforcement (in some genera only weakly developed). Hp with large and sclerotised bladder-like part ‘c’ of the labyrinth, postlabyrinthal internal spermiduct with up to 6 additional coils.

##### Tribes and genera included.

see Table 1.

#### ﻿Tribe Psychrodromini

Taxon classificationAnimaliaPodocopidaCyprididae

Martens, 2001

006AC914-8AAE-56D7-89F4-06184D32CD54

##### Diagnosis

**(adapted from Martens 2001).** Mostly compact carapace, with inwardly displaced selvage present or absent, fused zones without marginal septa. Rome organ on Al small or medium-sized, consisting of one or two parts. Second segment of Mx1 palp cylindrical (rectangular in the dissection), with length 1.5–2.5× the distal width. Seta d_l_ on T2 2–4× longer than seta d_2_. CR symmetrical or slightly asymmetrical, with proximal seta absent or shaped as a spine. Hp with 0–6 additional postlabyrinthal coils of the spermiduct; sometimes with a hook-like structure on the medial shield.

##### Genera allocated.

*Caledromus* gen. nov. (here allocated); *Humphcypris* Martens, 1997; *Psychrodromus* Danielopol & McKenzie, 1977; *Somalicypris* Martens, 1977.

##### Remarks.

The character “additional postlabyrinthal coils of the inner spermiduct in the Hp” refers to coils that cover most of the internal parts of the Hp, including the labyrinth, before the normal ventral coils which lead to the *bursa copulatrix*; see Martens (1997) for examples in *Humphcypris* and *Somalicypris*. For a discussion on the altered position of *Paranacypris*Higuti et al., 2009: see below. The above diagnosis therefore deviates from that in Higuti et al. (2009).

#### ﻿Genus Caledromus gen. nov.

Taxon classificationAnimaliaPodocopidaCyprididae

AF62BE1B-346B-58A9-A1F9-28F44685E6CB

https://zoobank.org/AD39BBD4-6C7E-4AF8-B595-3A06A533A9CF

##### Type species.

*Caledromusrobinsmithi* gen. et sp. nov.

##### Etymology.

*Cale* from the New Caledonian archipelago and *dromus* to indicate the close relationship to the genus *Psychrodromus*.

##### Diagnosis.

A genus typical of the Psychrodromini and closely related to *Psychrodromus*. Cp in dorsal view compact and rather wide, LV overlapping RV on all sides. A1 with small Rome-organ consisting of one segment only. A2 with natatory setae reduced to setulae or fully absent. Mx1-palp with second segment cylindrical (rectangular in slide), with L longer than basal W. T1 in female with broad palp, distally skewed and set with three very short setae. T2 with seta d_1_ ~ 2× the length of seta d_2_. CR symmetrical, proximal seta Sp an unmoveable spine. Hp without extra coils of the inner postlabyrinthal spermiduct.

The genus is presently monospecific and endemic to New Caledonia.

#### ﻿ Caledromus robinsmithi

Taxon classificationAnimaliaPodocopidaCyprididae

gen. et sp. nov.

D9575666-6197-5F72-8641-9BAB709A704B

https://zoobank.org/4C16B7A5-2500-4831-ABD7-BC31AD44B0D2

**Figure 2.**
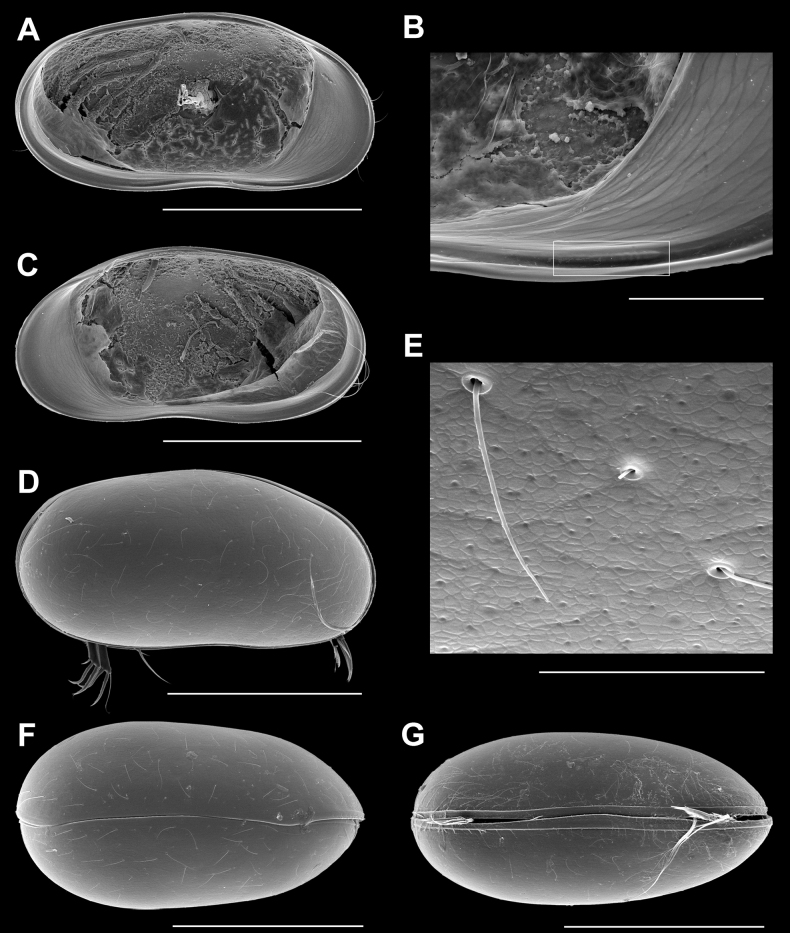
*Caledromusrobinsmithi* gen. et sp. nov., male **A**LVi (MNHN-IU-2023-181) **B**LVi, showing detail of anteroventral peg (in white frame) (MNHN-IU-2023-183) **C**RVi (MNHN-IU-2023-181) **D** CpRl (RBINS-INV-197990) **E** CpRl, detail of carapace surface of Fig. 2D**F**CpD (MNHN-IU-2023-184) **G**CpV (MNHN-IU-2023-185). Scale bars: 500 µm (**A, C, D, F, G**); 100 µm (**B**); 40 µm (**E**).

**Figure 3.**
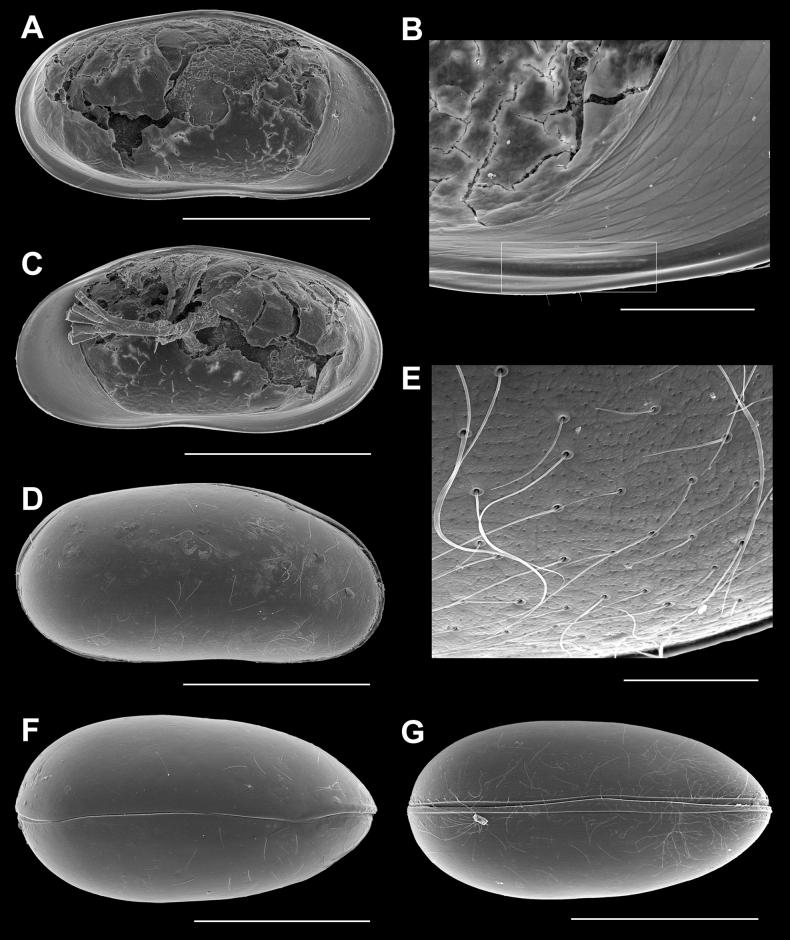
*Caledromusrobinsmithi* gen. et sp. nov., female **A**LVi (MNHN-IU-2023-182) **B**LVi, detail of anteroventral peg (white frame) (MNHN-IU-2023-186) **C**RVi (MNHN-IU-2023-182) **D** CpRl (RBINS-INV-197992) **E** CpRl, detail of carapace surface of Fig. 3D (RBINS-INV-197991) **F**CpD (MNHN-IU-2023-187) **G**CpV (MNHN-IU-2023-188). Scale bars: 500 µm (**A, C, D, F, G**); 100 µm (**B**); 50 µm (**E**).

**Figure 4.**
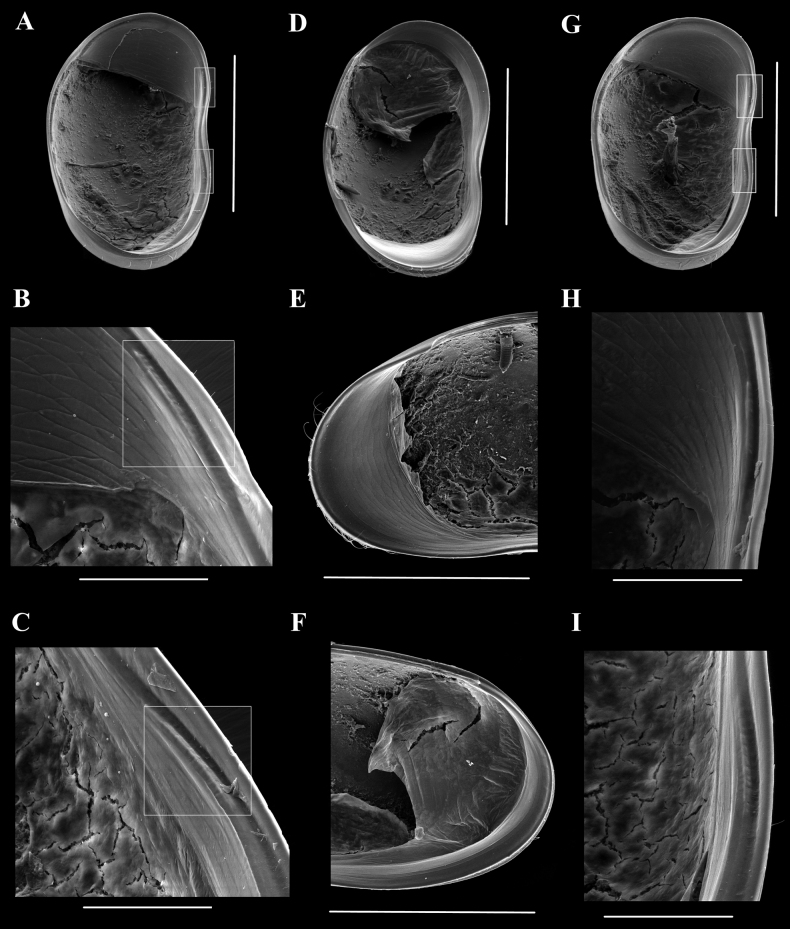
*Caledromusrobinsmithi* gen. et sp. nov. **A–F** female **G–I** male **A**LVi, photographed at tilted angle, showing inner anatomy of anterior side (MNHN-IU-2023-186) **B**LVi, detail of antero-ventral margin, showing peg (white frame) (MNHN-IU-2023-186) **C**LVi, detail of postero-ventral margin, showing peg (white frame) (MNHN-IU-2023-186) **D**RVi, photographed at tilted angle, showing inner anatomy of posterior side (MNHN-IU-2023-186) **E**RVi, detail of anterior margin (MNHN-IU-2023-186) **F**RVi detail of posterior margin (MNHN-IU-2023-186) **G**LVi (MNHN-IU-2023-181), photographed at tilted angle, showing inner anatomy of anterior side **H**LVi detail of antero-ventral margin, showing peg **I**LVi detail of postero-ventral margin, showing peg. Scale bars: 500 μm (**A–D, G–I**); 400 μm (**E, F**).

**Figure 5.**
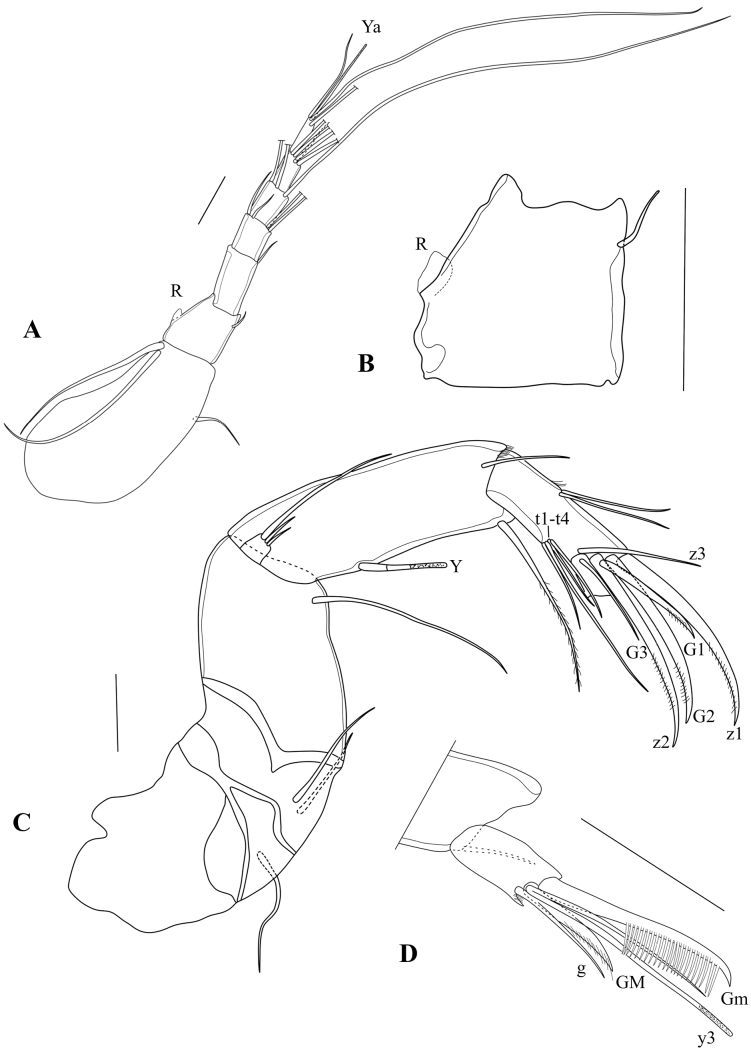
*Caledromusrobinsmithi* gen. et sp. nov., male **A**A1 (MNHN-IU-2023-183) **B**A1 detail of Rome Organ (MNHN-IU-2023-183) **C**A2 (MNHN-IU-2023-183) **D**A2 terminal segment (MNHN-IU-2023-183). Scale bars: 50 μm (**A–D**).

Figs 1 , 2 , 3 , 4 , 5

##### Type locality.

New Caledonia • North Province, Village of Poindimié in the area of the valley of the Necaapwé (sample HYNC2569). Coordinates: 20°57.165'S, 165°21.71'E. Altitude: 35 m. Collected on 28.05.2018. Road-side pool. Leg.: JH and KM. Holotype, allotype and paratypes all from the type locality (Fig. 1, Suppl. material 1).

##### Type material.

***Holotype*** • 1 ♂ (adult); dissected and stored on a permanent microscopic slide and valves stored dry in a micropalaeontological slide (MNHN-IU-2023-181).

***Allotype*** • 1 ♀ (adult); dissected and stored as the holotype (MNHN-IU-2023-182).

***Paratypes*** • 3 adult ♂♂ Cp (RBIN-INV-197990, MNHN-IU-2023-184, MNHN-IU-2023-185) and 3 adult ♀♀ Cp (RBIN-INV-197992, MNHN-IU-2023-187, MNHN-IU-2023-188) used for SEM. 1 adult ♂ (MNHN-IU-2023-183) and 2 adult ♀♀ (MNHN-IU-2023-186, RBIN-INV-197991) dissected and stored as the holotype. Several adult and juvenile ♂♂ and ♀♀ stored *in toto* in EtOH (MNHN-IU-2023-189).

##### Repositories.

Muséum national d’Histoire naturelle, Paris, France (MNHN-IU-2023-181-189) and Royal Belgian Institute of Natural Sciences, Brussels, Belgium (RBINS-INV. 197989-197992).

##### Etymology.

the species is named after Dr Robin Smith (Kusatsu, Shiga, Japan), in recognition of his significant contribution to research on non-marine ostracods, especially on those of the ancient Lake Biwa, and with much appreciation of years of friendship with KM.

##### Diagnosis.

Cp robust and rather wide in dorsal view. LV overlapping RV on all sides, but especially along posterio-dorsal and entire anterior side. Valve surface not striated, but set with random grooves, small pits, and rimmed pores around stiff setae. RV with inner list, LV with posterior marginal selvage. A1 with Rome-organ small, consisting of one segment. A2 with five natatory setae fully absent, only accompanying seta present; male with claw GM with hyper-developed row of spines. Md-palp with alpha and beta setae both short and elongated. Mx1-palp with second segment cylindrical (rectangular in the dissection), with L ~ 1.5× basal width. T1 in female with large, inflated, and asymmetrical palp, distally with three short setae; setae b, d and a present, seta d longer in the male than in the female. Prehensile palps in male both with sickle-shaped second segment, in Rpp longer than in Lpp. T2 with seta d_1_ ~ 2× as long as d_2_. T3 with a distal pincer-shaped organ. CR without sexual dimorphism, with robust ramus, seta Sp an unmoveable claw, seta Sa longer than claw Ga, the latter almost straight, claw Gp distally curved. Attachment of CR with proximal triangular re-enforcement, branch vb almost straight, branch db curved backwards. Hp with asymmetrically rounded ms without hook-like expansion; ds consisting of two parts, ds1 rectangular and distally situated, ds2 rounded and situated mid-dorsally; internal anatomy without additional coils of the postlabyrinthal spermiduct. Zenker organ elongated, with ~ 20 coils.

##### Description.

**Male.**Cp in lateral view (Fig. 2D) elongated, with anterior margin more broadly rounded than the posterior one; greatest height situated anterior to the middle; LV overlapping RV along all sides, but specifically along the antero-dorsal and the anterior side and with a weakly rounded ventral flap. CpD (Fig. 2F) with W ~ 1/2 of the L, greatest width situated in the posterior ¼; posterior edge more broadly rounded than anterior one. CpV (Fig. 2G) showing both valves with straight ventral outer lists. Valve surface not striated, but set with random grooves, small pits, and rimmed pores around stiff setae (Fig. 2E).

LVi (Fig. 2A, B) with anterior margin more broadly rounded than posterior one, greatest height at ~ 1/3 from the anterior side, dorsal margin from that point onwards sloping towards the less broadly rounded posterior margin, ventral margin weakly curved; anterior calcified inner lamella broad, posterior one very narrow; inner list running briefly along the anterior margin, robustly along the ventral margin forming a groove and showing antero-ventral and postero-ventral shallow pegs (Figs 2B, 4G–I).

RV (Fig. 2C) with shape similar that that of LV, but with anterior margin more produced and with submarginal selvage running along most of the anterior, ventral, and posterior margins.

A1 (Fig. 5A, B) with seven segments. First segment with two long ventro-apical setae, and one short mid-dorsal seta; Wouters’ organ not seen. Second segment sub-quadrate, distally narrowing, with one sub-apical seta; Rome organ small, consisting of one rounded segment (Fig. 5B). Third segment rectangular, slightly > 2× as long as basal width, with one short dorsal seta. Fourth segment almost as long as wide, with two long dorsal setae and two unequal, much shorter ventral setae. Fifth segment slightly longer than wide, with two long ventral and one long dorsal natatory setae. Sixth segment slightly longer than wide, with four long and one short natatory setae. Terminal (7^th^) segment ~ 2× as long as wide, with single shorter seta, one aesthetasc Ya of sub-equal length, and two long natatory setae.

A2 (Fig. 5C, D) with exopodite reduced to a small plate, bearing one long and two short setae. Endopodite 3-segmented. First segment elongated and stout, aesthetasc Y long and slender (~ 1/3 of length of segment); five natatory setae fully absent, accompanying (6^th^) seta just reaching halfway of second endopodal segment. Second endopodal segment with two dorso-lateral and four ventro-lateral (t-) setae; of the latter, seta t_2_ ~ 2× as long as the other three setae; distal chaetotaxy typical of male Cyprididae, with clear sexual dimorphism, consisting of four claws (z1 and z2 being the largest, G2 being slightly shorter, G1 very short), and two setae (z3 and G3). Terminal (3^rd^) endopodal segment (Fig. 5D) with large claw Gm bearing a row of ~ 20 hyper-developed spines, a shorter but stout claw GM, a short seta g and long aesthetasc basally fused with a long, but slightly shorter seta.

**Figure 6.**
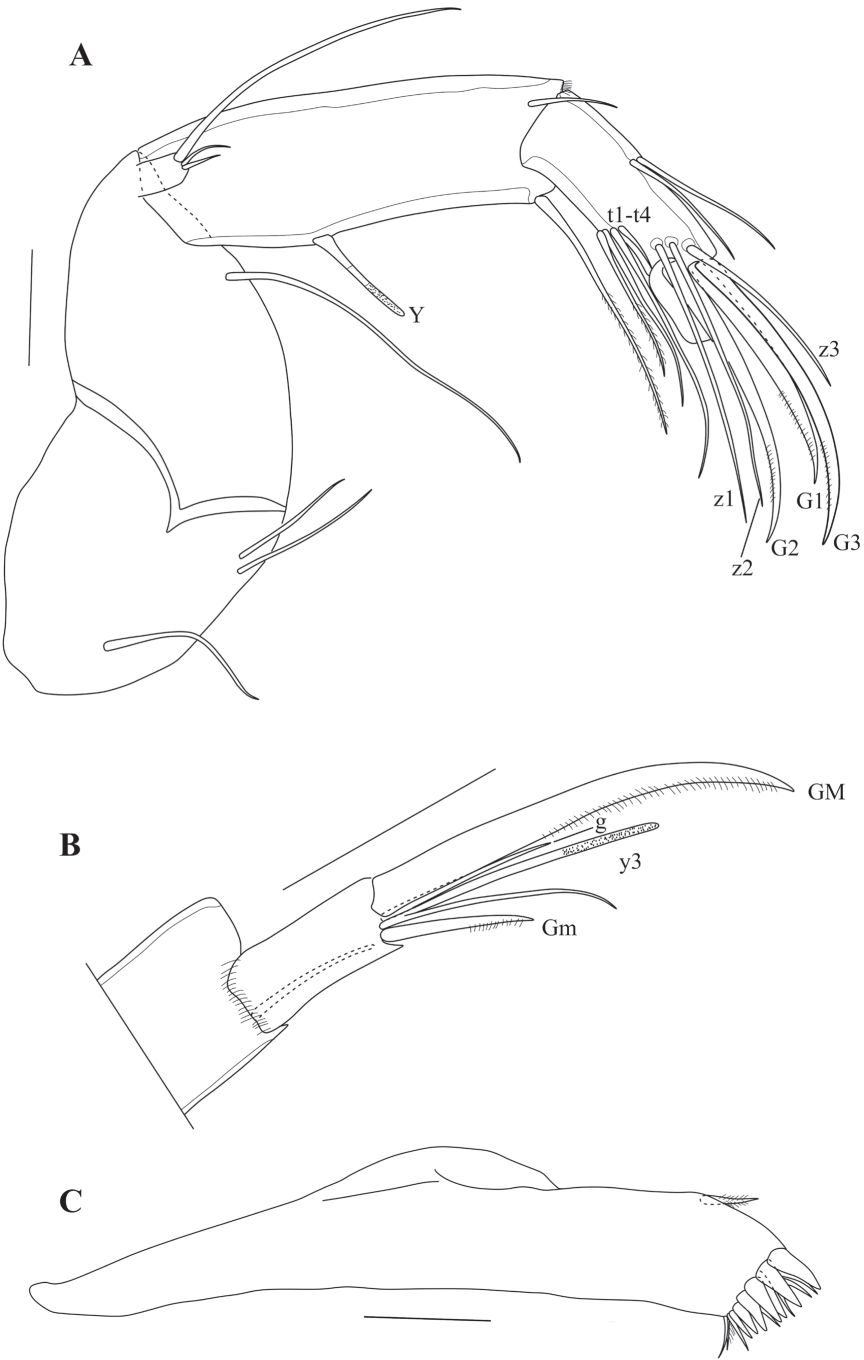
*Caledromusrobinsmithi* gen. et sp. nov. **A, B** female **C** male **A**A2 (MNHN-IU-2023-186) **B**A2 terminal segment (MNHN-IU-2023-186) **C**Md-coxa (MNHN-IU-2023-181). Scale bars: 50 μm (**A–C**).

**Figure 7.**
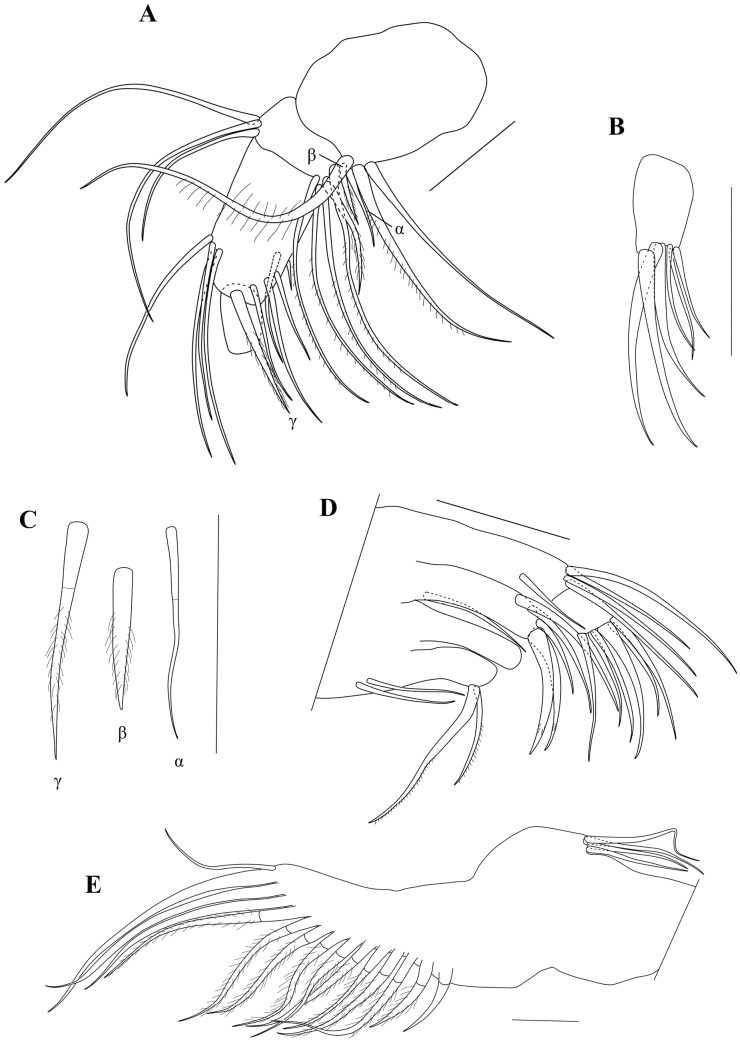
*Caledromusrobinsmithi* gen. et sp. nov. ♂ **A**Md-palp (respiratory plate not illustrated) (MNHN-IU-2023-181) **B**Md-palp last segment (MNHN-IU-2023-181) **C**Md-palp, alfa, beta, and gamma setae (MNHN-IU-2023-181) **D**Mx1, palp and three endites (MNHN-IU-2023-181) **E**Mx1, respiratory plate (MNHN-IU-2023-181). Scale bars: 50 μm (**A–E**).

Md with coxal plate (Fig. 6C) elongated, distally set with rows of spines and small setae. Md-palp (Fig. 7A–C) with alpha-seta rather long, slender, and smooth; beta-seta shorter, ~ ¾ the length of the alpha seta, sender and hirsute; gamma-seta large, broad and hirsute in distal 2/3 of its length. First segment with two long barbed setae s1 and s2), one long smooth seta and the alpha seta. Second segment dorsally with a group of three smooth setae (two long, one shorter), ventrally with four long and hirsute and one shorter hirsute setae as well as the beta seta. Third segment dorsally with four subapical and subequal setae, ventrally with one subapical seta and a short aesthetasc, medially with four setae (three plus gamma-seta). Terminal segment (Fig. 7B) with three slender claws and three long setae.

**Figure 8.**
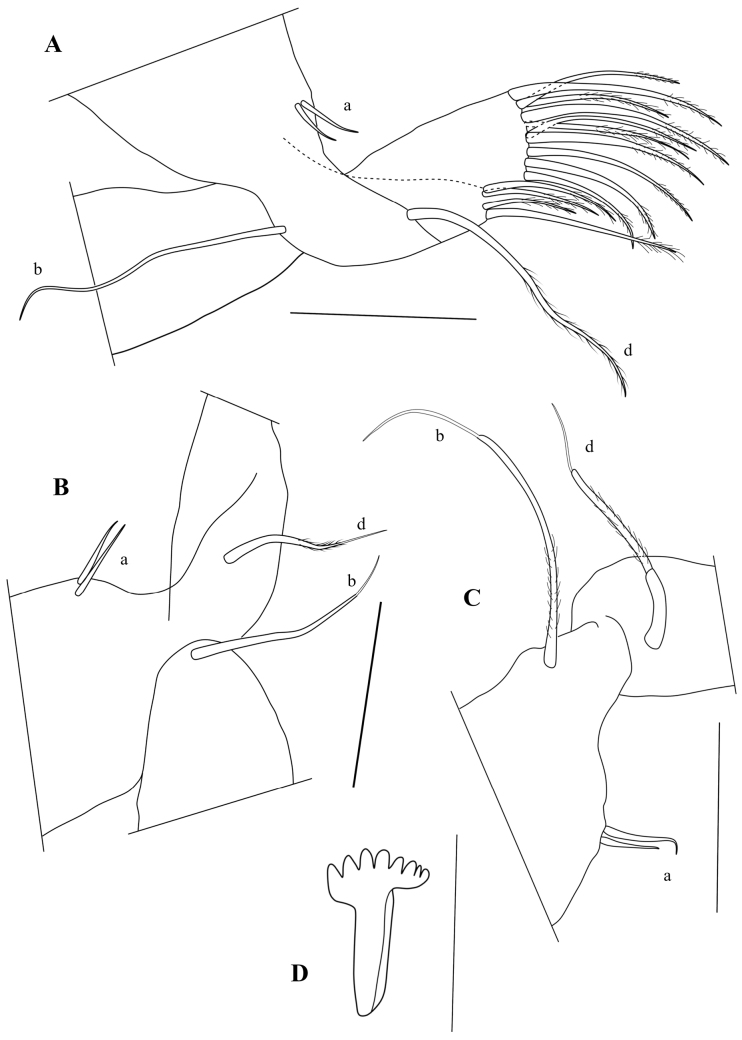
*Caledromusrobinsmithi* gen. et sp. nov. ♂ **A**T1, without prehensile palps (see Fig. 11) (MNHN-IU-2023-181) **B**T1, without palp, showing short setae b and d (MNHN-IU-2023-183) **C**T1, other side, without palp, showing long setae b and d with aberrant distal morphology (MNHN-IU-2023-183) **D** Rake-like organ (MNHN-IU-2023-183). Scale bars: 50 μm (**A–D**).

Rake-like organ (Fig. 8D) of normal shape, with ~ 8 unequal teeth.

**Figure 9.**
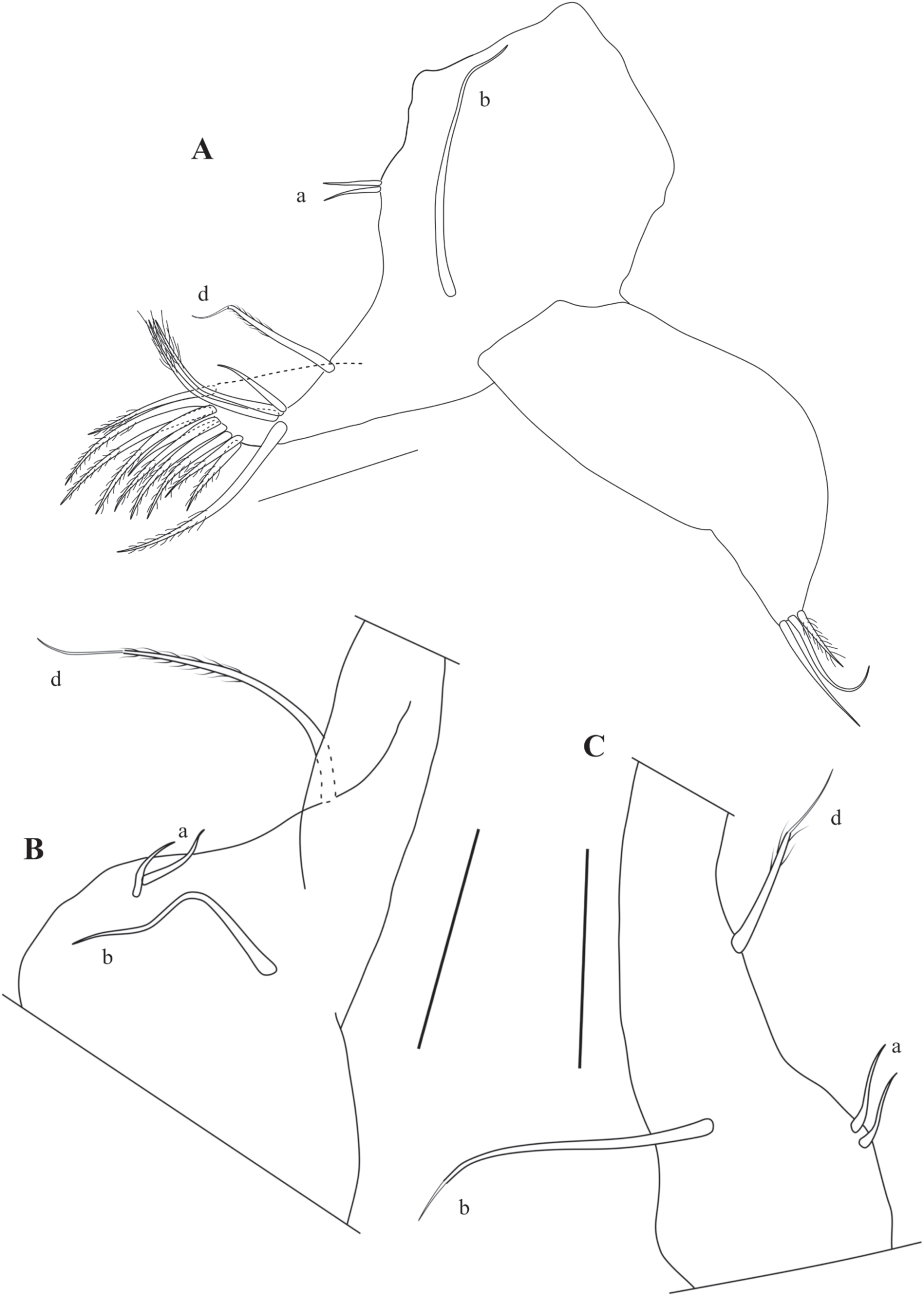
*Caledromusrobinsmithi* gen. et sp. nov. ♀ **A**T1 (MNHN-IU-2023-182) **B**T1, without palp, showing long seta d and short seta b (RBINS-INV-197991) **C**T1, other side, without palp, showing short seta d and long seta b (RBINS-INV-197991). Scale bars: 50 μm (**A–C**).

Mx1 (Fig. 7D, E) with second palp-segment cylindrical (rectangular in slide), L ~ 1.5× W; two *Zahnborsten* on third endite, one more robust, one more slender, both smooth. Sideways directed bristles unequal: one 2× as long as the second, the latter short and more slender, both hirsute. Respiratory plate (Fig. 7E) large and elongate, distally with a row of ~ 14 hirsute rays of distally increasing length, apically with one short smooth seta; proximally with a bundle of four smooth setae of medium length.

T1 (Figs 8A–C, 11C, D) with distal chaetotaxy of coxal plate consisting of ten setae of different shape and length and a group of four subapical setae, one longer, two of medium length and one shorter; proximally with two short a-seta; setae d and b showing left/right asymmetry: in T1 on one side both setae d and b relatively short, with b still longer than d, the latter only ~ 2× the length of the a-setae (Fig. 8B); in the other T1 (Fig. 8C) setae b and d almost 2× as long, b still longer than d (in the specimen illustrated here, both setae b and d appear to end in a flagellum, but this is not so in all specimens). Prehensile palps (Fig. 11C, D) slightly asymmetrical; first segments of sub-triangular shapes, with a large ventral protuberance, set with two large subequal sensory organs; second segments sickle shaped, in Rpp (Fig. 11C) narrower and more elongated than in Lpp (Fig. 11D), distally with one long and stout sensory organ.

**Figure 10.**
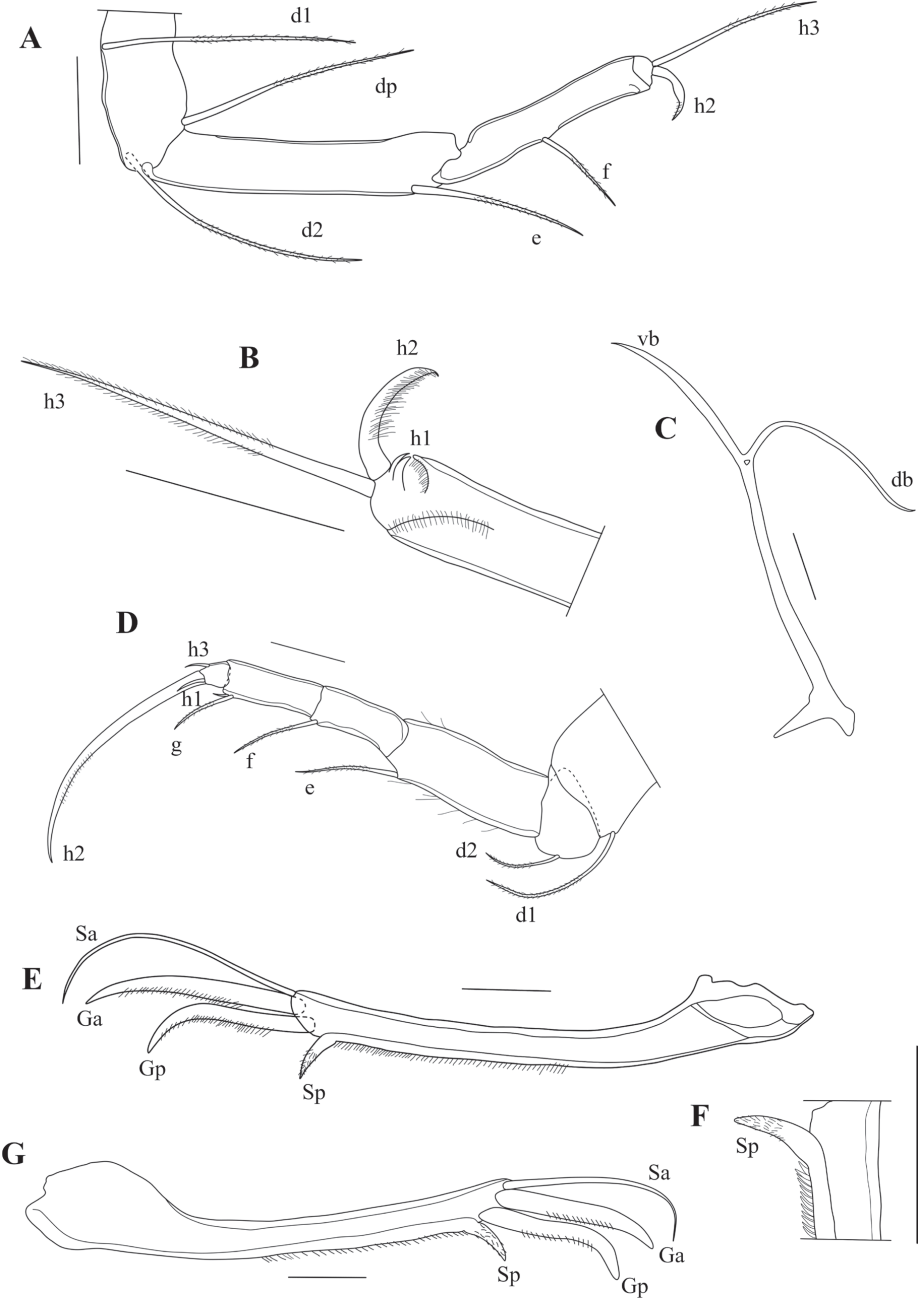
*Caledromusrobinsmithi* gen. et sp. nov. **A–F** ♂ **G** ♀ **A**T3 (MNHN-IU-2023-181) **B**T3, detail of last segment (MNHN-IU-2023-181) **C**Att of CR (MNHN-IU-2023-181) **D**T2 (MNHN-IU-2023-183) **E**CR (MNHN-IU-2023-183) **F**CR detail (MNHN-IU-2023-183) **G**CR, other side (MNHN-IU-2023-182). Scale bars: 50 μm (**A–E, G**); 25 μm (**F**).

T2 (Fig. 10D) with elongated segments and relatively long end claw. First segment with seta d1 long, reaching beyond second (knee) segment, > 2× as long as seta d2. Third segment with a long ventro-apical e-seta, almost reaching tip of fourth segment. Fourth segment divided into two elongated sub-segments: segment 4a with a ventro-apical seta f, not reaching tip of segment 4b, this segment with a ventro-apical seta g. Fifth segment with an apical seta h1, a subapical seta h3, and a long and thin apical claw h2.

T3 (Fig. 10A, B) a cleaning limb. First segment with three long setae, ventrally with setae d1 and d2, dorsally with seta dp. Second segment very elongated, with L ~ 5× basal W, with a long subapical seta e. Third segment with a shorter lateral seta f, apical seta g missing. Distal part of 3^rd^ segment and 4^th^ segment fused to a pincer shaped organ, bearing one long seta h3, one broadly and a curved hirsute seta h2 of medium length. Pincer organ (Fig. 10B) with spine, minute seta h1 and flanked by rows of setulae.

CR (Fig. 10E–G) symmetrical, with slightly curved, robust ramus, ventrally set with a row of setulae; seta Sp transformed in a stout, unmoveable spine (Fig. 10F); claw Gp robust and distally curved, claw Ga robust and straight, seta Sa a real seta, significantly longer than claw Ga.

Att (Fig. 10C) with simple distal bifurcation and solid triangular structure at basis (the latter typical of all Herpetocypridinae).

**Figure 11.**
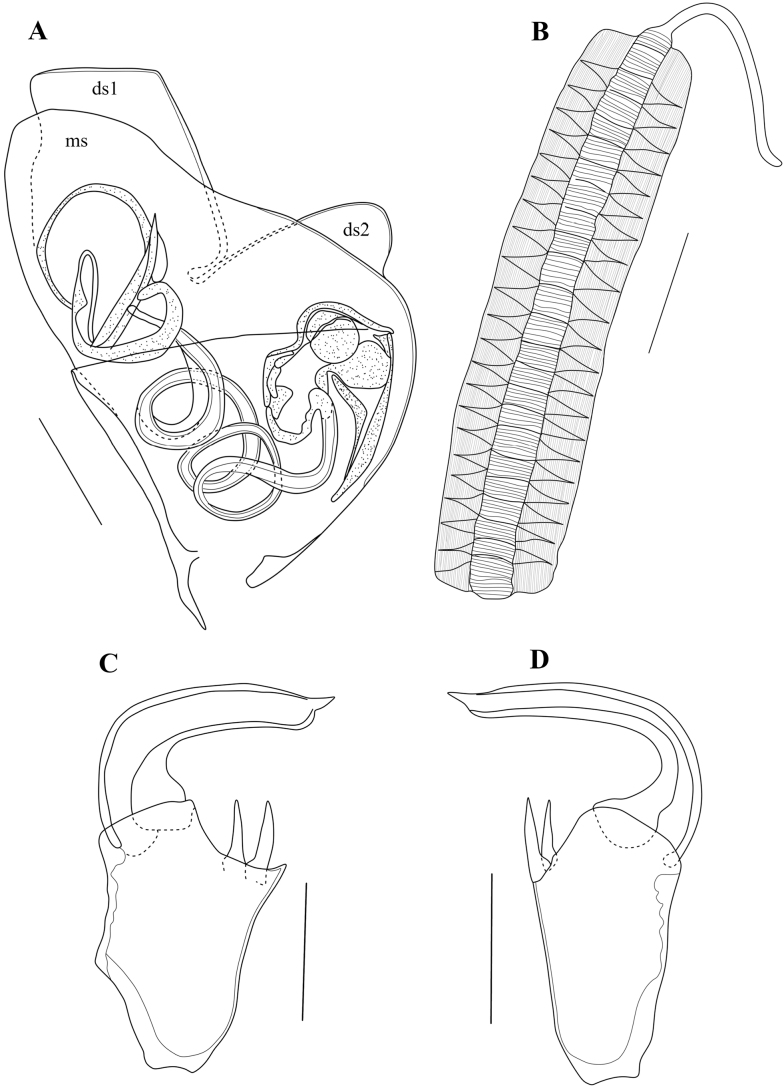
*Caledromusrobinsmithi* gen. et sp. nov. ♂ **A**Hp (MNHN-IU-2023-181) **B** Zenker Organ (MNHN-IU-2023-181) **C**Rpp (MNHN-IU-2023-183) **D**Lpp (MNHN-IU-2023-183). Scale bars: 50 μm (**A–D**).

Hp (Fig. 11A) well-sclerified, consisting of an asymmetrically rounded ms without hook-like expansion; ds consisting of two lobes, ds1 rectangular and distally situated, ds2 rounded and situated mid-dorsally; internal anatomy without additional coils of the postlabyrinthal spermiduct (see above); three ventral coils leading to bursa copulatrix well developed.

Zenker organ (Fig. 11B) elongated, with ~ 20 coils.

**Female** (only sexual dimorphism with males given). Cp (Fig. 3D–G), RV (Figs 3C, 4D–F), LV (Figs 3A, B, 4A–C), A1, Md, Mx1, T2, T3, CR and CR-attachment as in the male.

A2 (Fig. 6A, B) with distal chaetotaxy of second endopodal segment consisting of three G-claws and three z-setae; claws G1 and G3 the longest and subequal, setae z1 and z2 the longest and subequal, seta z3 the shortest. Terminal (3^rd^) endopodal segment with claw GM long and stout, but without the hyper-developed spines.

T1 (Fig. 9A–C) with distal chaetotaxy of coxal plate consisting of ten setae of different shapes and lengths, and a group of four subapical setae, one proximal seta significantly longer, two of medium length and one shorter; proximally with two short a-seta; setae d and b showing left/right asymmetry: T1 on the one side with seta d much longer and seta b relatively short, ~ 2–2.5× length of setae a (Fig. 9B); in the other T1 (Fig. 9C) seta d shorter and set b much longer. Palp wide and large, distally slightly skewed to one side, set with three very short and slightly unequal setae (Fig. 9A).

Female copulatory organs (not illustrated) as typical of the subfamily, i.e., rounded, slightly elongated structures with few visible internal or external features.

##### Measurements.

See Table 2.

**Table 2.** *Caledromusrobinsmithi* gen. et sp. nov., measurements of illustrated specimens.

Slide/specimen nr	Valve/Cp	M/F	L (μm)	H (μm)	W (μm)
MNHN-IU-2023-183	LV	M	925	456	
MNHN-IU-2023-183	RV	M	897	448	
MNHN-IU-2023-181-HOLOTYPE	LV	M	908	451	
MNHN-IU-2023-181-HOLOTYPE	RV	M	882	447	
RBINS-INV-197989	LV	M	985	491	
RBINS-INV-197989	RV	M	968	485	
RBINS-INV-197990	CpRL	M	931		466
MNHN-IU-2023-184	CpD	M	921		497
MNHN-IU-2023-185	CpV	M	907		456
MNHN-IU-2023-186	LV	F	997	485	
MNHN-IU-2023-186	RV	F	975	482	
RBINS-INV-197991	LV	F	971	490	
RBINS-INV-197991	RV	F	955	482	
MNHN-IU-2023-182-ALLOTYPE	LV	F	1023	510	
MNHN-IU-2023-182-ALLOTYPE	RV	F	989	502	
RBINS-INV-197992	CpRL	F	992		485
MNHN-IU-2023-187	CpD	F	1035		535
MNHN-IU-2023-188	CpV	F	976		503

##### Ecology.

The species is presently known from its type locality only: a shallow roadside pool, with water coming from a small stream; with terrestrial plants, grasses and “Songe” on sediment consisting of gravel and mud (Suppl. material 1). Depth: 0.05–0.1 m. Water temperature: 21 °C. Electrical conductivity: 103 µS/cm. pH: 6.64.

## ﻿Discussion

### ﻿Homeomorphic characters

Every taxonomic hierarchy is a working hypothesis, to be tested by using new characters or new methods, or by the discovery of new taxa. Especially in cases where different characters show different distribution patterns in taxa, it can be difficult to determine which characters show the correct phylogeny and which are homeomorphic (see for example, discussion in Almeida et al. 2023). Phylogenetic analyses, based on large sets of either morphological or molecular characters, or on both, are the best way to minimise subjective decisions, but such analyses are not always possible. A good example of a potential convergent evolution in the Herpetocypridinae is in the tribe Isocypridini. One of the morphological characters defining the genus *Amphibolocypris* is the claw-like subapical seta on the distal segment of the walking leg (T2), so that species in this genus have two distal claws on the T2. The exclusively southern African *Amphibolocypris* shares this character with the genus *Platycypris*, which is endemic to Australia (De Deckker 1981a). This shared character could be the result of common descent. However, it is also possible that *Amphibolocypris* and *Platycypris* are less closely related than it would seem, because a double end claw on T2 also occurs in other, unrelated genera, for example in *Limanocypris* Schornikov, 1961 (Limanocypridinae Hartmann & Puri, 1974) from Siberia, the Spanish endemic *Candelacypris* Baltanás, 2001 (Eucypridinae Bronshtein, 1947) and in *Scottia* Brady & Norman, 1889 (Scottiinae Bronshtein,1947). Here, we provide a brief overview of the major characters and their character states used in the taxonomic hierarchy of the Herpetocypridinae.

### ﻿Characters used in the taxonomy of the Herpetocypridinae

The most important synapomorphy to unite all Herpetocypridinae is the presence of a supporting triangular chitinous structure at the base of the CRAtt (Triebel 1953; Martens 2001). Of course, this is only a workable character for Recent taxa, so we here refer to Danielopol et al. (1986) for features that allow the identification of fossil Herpetocypridinae.

The marginal valve anatomy, including the width of the fused zone, the morphology of the marginal pore canals (single or branched), the presence of false pore canals (as in *Herpetocypris*), and generally the presence or absence and the morphology of selvages and inner lists are important characters which define genera and tribes. The fact that valves in this group are bearing such clear identifiable characteristics also allows fossils to be readily identified (Danielopol et al. 1986). The largely inwardly displaced selvage and the wide fused zone in the LV of *Herpetocypris* is a case in point, and the distinctively raised, inwardly displaced selvage at the postero-ventral part of the RV in *Thaicypris* even more so. Several genera in the Stenocypridini, and the genus *Stenocypris* most of all, have clear marginal septa along at least the anterior valve margin, which is again a useful character for both neontologists and palaeontologists. It was this feature which allowed Horne and Martens (1998) to recognise some Mesozoic fossils, e.g., *Mantellianamantelli* (Anderson, 1971), as being most likely a species of the extant genus *Stenocypris*. However, also other genera have marginal septa, e.g., species of the distantly related *Cypretta* Vara, 1895 (Cyprettinae, Hartmann, 1963) and in *Pseudocypretta* Klie, 1932 (Cypridopsinae, Kaufmann, 1900). Shearn et al. (2014) discovered a new character within the LV of *Ilyodromus* species, namely the presence of one or two ventral inner pegs, as remnants or fortifications of an ancient inner list. Here, we find similar, albeit less pronounced, structures in *Caledromus*, even though these genera are presently placed in different tribes (Herpetocypridini and Psychrodromini, respectively).

Within the Herpetocypridinae, the Rome Organ on the A1 has a plastic morphology. For example, within the Herpetocypridini, the genera *Candonocypris* and *Herpetocypris* have invariably small Rome organs. Amongst species of *Ilyodromus*, however, this organ can be longer than the actual segment on which it is situated and can be two- and even three-segmented. Most other genera in the Herpetocypridinae have small Rome Organs, but in *Ampullacypris* (Stenocypridini) even this small organ can be two-segmented (De Deckker 1981b).

The length of the natatory setae on the A2 can vary greatly, even between species of the same genus (see for example *Humphcypris*). However, except for *Caledromusrobinsmithi* where the five natatory setae have completely disappeared, in the other genera and species of the subfamily some small remnants always remain. Even in the presently described species without any trace of the natatory setae, the accompanying seta is still present and not reduced, which clearly shows that this seta has a developmental program which is unrelated to those of the actual natatory setae. The inverse situation occurs in *Brasilocypria*Almeida et al., 2023 (Cyclocypridinae) where the species have very long natatory setae, but where the accompanying seta has disappeared (Almeida et al. 2023).

In males of several species and genera of Herpetocypridinae (e.g., *Herpetocypris*, *Humphcypris*, *Somalicypris*, *Caledromus*, and others, but not in *Thaicypris*), the male A2 has a the larger claw Gm set with a row of long spines, which are absent in females. Some Cypridopsinae, e.g., the African *Zonocypris* G.W. Müller, 1898 and the Brazilian *Cabelodopsis* Higuti & Martens, 2012, have a similar morphology, but the hyperdeveloped claw occurs in females only. Smith et al. (2023) hypothesised that in the latter case these claws are used by the females to clean vegetal substrates for egg deposition. This is of course possible, but the present equivalent in male Herpetocypridinae most likely has a different function, maybe as part of mate recognition during copulation (see also discussion in Higuti and Martens 2012).

The shape of the second segment of the Mx1 palp is important in the taxonomy of the Herpetocypridinae, as species in the tribes Herpetocypridini and Isocypridini have spatulate segments, while species in the tribes Stenocypridini and Psychrodromini have cylindrical palp segment, which show up as rectangular structure in the dissections (see discussion on the position of the genus *Paranacypris* below).

The useful characters of the T1 are mostly those related to the morphology of the male prehensile palps, but also the presence/absence, shape and length of the setae d, b, and a can be useful in the taxonomy of several groups. In the present species, both males and females have asymmetrical setae b and d, with lengths varying between left and right sides. It is the first time that this is being reported in this group, but it is a feature which is rarely observed and described for both limbs of the same individual. The new genus *Caledromus* is characterised by the presence of a wide and broad female T1-palp with very small apical setae, an autapomorphic feature, and therefore useful to identify this taxon, but not helpful regarding taxonomic placement of the genus. Also, several other characters are autoapomorphic for one genus and not informative for phylogenetic studies. One example is the monospecific genus *Stenocypria*, where the sinuous posterior inner margin of the female valves of *S.fischeri* (Lilljeborg, 1883) results from a lobe-shaped expansion of the calcified inner lamella. This calcified lobe is absent in the males (Scharf et al. 2020). Another example is the presence of two f-setae on the T3 in the species of the genus *Candonocypris*. A similar, but unrelated, duplication of setae can be found in species of *Chlamydotheca* Saussure, 1858, where the T2 invariably has two e-setae (e.g., *Chlamydothecacolombiensis* Roessler, 1985).

The morphology of the female T1-palp in *C.robinsmithi* is interesting from an evolutionary point of view, as the morphology of this palp resembles that of the shape of prehensile palps in A-1 males. This could thus be a case of heterochronic development, of which several examples exist in non-marine ostracods, for example in the African and Australian terrestrial representatives of the subfamily Scottiinae Bronshtein, 1947 (see Martens et al. 2004).

The potential relevance of the presence/absence and length ratios of the d_1_ and d_2_ setae on the T2 for the identification at the genus level has already been stressed several times (see Martens 2007 for Cypridini, Martens et al. 1992 for Eucypridinae and others). In the present subfamily also, the application of this character proves to be useful, but again at the level of genus rather than at that of tribes.

The differences in the morphology of the seta Sa on the CR (with character states: simple seta, moveable claw-like seta, immobile spine, fully absent) were amongst the first reasons that drove Danielopol and McKenzie (1977) to distinguish between *Ilyodromus* and their new genus *Psychrodromus*. This distinction stands till the present and is now valid at the level of the tribes of the Herpetocypridinae, as for example, the above two genera belong to two different tribes.

The Hp, in species and genera in which males are known, comprises useful taxonomic information. Mostly, external Hp morphology can be used to identify species within genera. Here, however, the morphology of the inner spermiduct, with between 0 (in *Caledromus*) and 6 (in *Somalicypris*) additional postlabyrinthal coils (see definition above) allows divisions even at the level of tribes (see diagnoses in Martens, 2001). The illustrated Hp of *Thaicypris* (in Savatenalinton 2022c) has a weakly developed inner anatomy, which indicates that the male specimen might have been sub-adult. Nevertheless, the presence of a single additional coil of the postlabyrinthal spermiduct is visible. Finally, also the presence/absence of sclerotised hook-like structures on the ms of the Hp are taxonomically informative (in Psychrodromini present in all genera but *Caledromus*).

### ﻿Taxonomic position of *Caledromus*

Based on the set of characters described above and on the diagnoses of the four tribes in the Herpetocypridinae (see Martens 2001; Shearn et al. 2017b), *Caledromus* is herewith lodged in the Psychrodromini, primarily because of the compact and broad Cp in dorsal view, the absence of marginal septa on the valves, the cylindrical (not spatulate) second palp segment of the Mx1-palp with L ~ 1.5× W, the long seta d_1_ (d_1_ = ~2 × d_2_), and the symmetrical CR with an immobile spine Sp. The genus differs from all three other genera in the tribe by the complete absence of additional postlabyrinthal coils in the Hp, the complete absence of the natatory setae on the A2, and the broad, asymmetrical T1-palp with minute apical setae in the female.

### ﻿Taxonomic position of *Paranacypris*

Higuti et al. (2009) lodged *Paranacypris* in the tribe Psychrodromini of the Herpetocypridinae, based on several characters which showed a close relationship with the genus *Psychrodromus*. For this, they had to deal with two major characters in their genus which deviated from the diagnosis of this tribe. Firstly, the complete disappearance of seta d_2_ on T2. Higuti et al. (2009) argued that in the Psychrodromini this seta was already reduced when compared to seta d_1_, so that the complete disappearance was just one evolutionary step further. Secondly, the spatulate second segment of the Mx1-palp, which is typical of the Herpetocypridini, was considered a result of mosaic evolution. The diagnosis of the tribe Psychrodromini was adapted to allow inclusion of *Paranacypris*. Here, based on the discovery and relevance of *Caledromus*, we reconsider this decision and now lodge *Paranacypris* in the Herpetocypridini. This is based on its closer relationship with *Ilyodromus*, namely because of the spatulate second segment of the Mx1-palp, and the long Rome Organ on the A1 (Table 1). The stout gamma-seta on the Md-palp stands as a character which is not decisive for either of the two tribes.

### ﻿Ostracod fauna of New Caledonia

Although several ostracod species from the New Caledonian expeditions still await description, the ones that were reported from this archipelago (see table in Kisseih et al. 2020) already provide some ideas on the zoogeographical affinities of its ostracod fauna. The species of *Stenocypris* and *Strandesia*, *Cyprinotusdrubea*Martens et al., 2019 and *Cyprisgranulata* Daday, 1910 can already qualify as belonging to circumtropical lineages. *Kennethiamajor* (Méhes, 1939) clearly has Australian affinities. The same is true for *Candonocyprisnovaezelandiae* (Baird, 1843), but this species has meanwhile “conquered the world” as a successful alien invasive species (see Scharf et al. 2014). With *Caledromusrobinsmithi*, however, we have a species and genus which shows, because of its affinity with *Psychrodromus*, more pronounced Palaearctic connections. Therefore, it seems that the geographical position of the New Caledonian archipelago, isolated as this region may seem to be, has been prone to invasions by lineages with different zoogeographical origins. Further research on thus far undescribed radiations will further illuminate the geographical origin of the New Caledonian non-marine ostracods.

## Supplementary Material

XML Treatment for Herpetocypridinae

XML Treatment for Psychrodromini

XML Treatment for Caledromus

XML Treatment for Caledromus robinsmithi

## ﻿Additional information

### Author ORCIDs

Koen Martens https://orcid.org/0000-0001-8680-973X

Vitor Góis Ferreira https://orcid.org/0000-0002-5244-7707

Nadiny Martins de Almeida https://orcid.org/0000-0002-4380-125X

Janet Higuti https://orcid.org/0000-0002-3721-9562

### Data availability

All of the data that support the findings of this study are available in the main text or Supplementary Information.

## Supplementary materials

Supplementary material 1 Type locality of *Caledromusrobinsmithi* gen. et sp. nov. Data type (measurement/occurrence/multimedia/etc.) https://binary.pensoft.net/file/858073This dataset is made available under the Open Database License (http://opendatacommons.org/licenses/odbl/1.0/). The Open Database License (ODbL) is a license agreement intended to allow users to freely share, modify, and use this Dataset while maintaining this same freedom for others, provided that the original source and author(s) are credited.Koen Martens, Vitor Góis Ferreira, Nadiny Martins De Almeida, Janet Higuti
