# Supplementary figures and images for: ﻿On Caledromusrobinsmithi, a new genus and species of Psychrodromini Martens, 2001 (Crustacea, Ostracoda, Herpetocypridinae) from New Caledonia (Pacific Ocean)
Source: Zookeys. 2023 Jun 1;1165:155–82. doi: 10.3897/zookeys.1165.104045 (PMC10251247; doi:10.3897/zookeys.1165.104045)

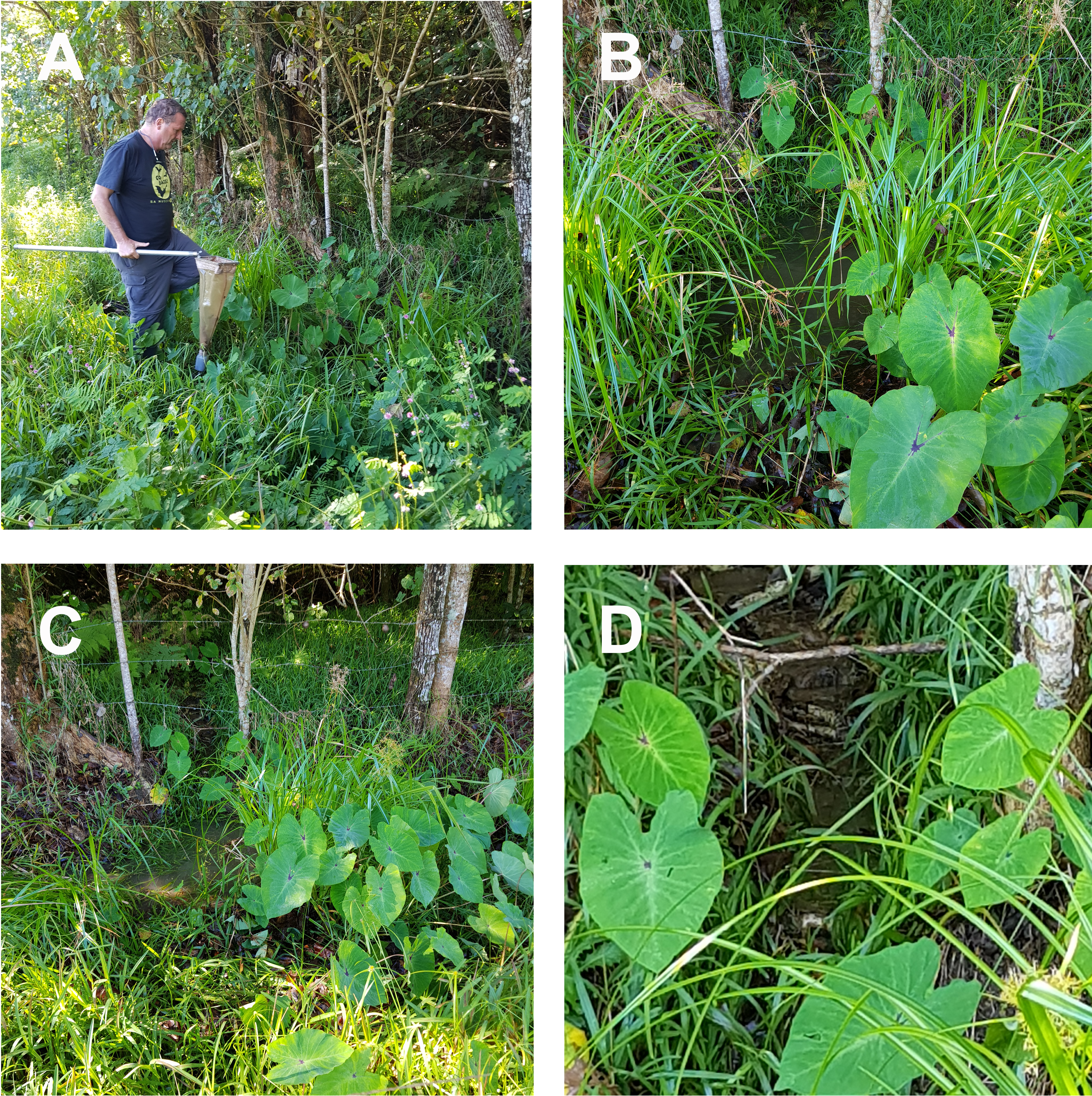

Supplement: Supplementary material 1 — Type locality of Caledromusrobinsmithi gen. et sp. nov. [file zookeys-1165-155_article-104045__-s001.tif]
